# VEGF Axonal Transport Dependent on Kinesin-1B and Microtubules Dynamics

**DOI:** 10.3389/fnmol.2017.00424

**Published:** 2017-12-21

**Authors:** Ping Yang, Xiao Sun, Zeng-Wei Kou, Kun-Wei Wu, Ya-Lin Huang, Feng-Yan Sun

**Affiliations:** ^1^Department of Neurobiology, Institute for Biomedical Science and State Key Laboratory of Medical Neurobiology, School of Basic Medical Sciences, Shanghai Medical College of Fudan University, Shanghai, China; ^2^Research Center on Aging and Medicine, Fudan University, Shanghai, China

**Keywords:** neurovascular unit, neuropeptide, neuromodulator, axon transport, neurotrophin

## Abstract

Axon-transport plays an important role in neuronal activity and survival. Reduced endogenous VEGF can cause neuronal damage and axon degeneration. It is unknown at this time if VEGF can be transported within the axon or whether it can be released by axonal depolarization. We transfected VEGF-eGFP plasmids in cultured hippocampal neurons and tracked their movement in the axons by live-cell confocal imaging. Then, we co-transfected phVEGF-eGFP and kinesin-1B-DsRed vectors into neurons and combined with immunoprecipitation and two-color imaging to study the mechanism of VEGF axon-trafficking. We found that VEGF vesicles morphologically co-localized and biochemically bounded with kinesin-1B, as well as co-trafficked with it in the axons. Moreover, the capacity for axonal trafficking of VEGF was reduced by administration of nocodazole, an inhibitor of microtubules, or kinesin-1B shRNA. In addition, we found that VEGF could release from the cultured neurons under acute depolarizing stimulation with potassium chloride. Therefore, present findings suggest that neuronal VEGF is stored in the vesicles, actively released, and transported in the axons, which depends on the presence of kinesin-1B and functional microtubules. These results further help us to understand the importance of neuronal VEGF in the maintenance of neuronal activity and survival throughout life.

## Introduction

Vascular endothelial growth factor (VEGF) is recognized as an important neuronal modulator, although it was originally identified as a key regulator of vascularization and angiogenesis (Ferrara and Henzel, 1989). VEGF emerged early during evolution of the invertebrate nervous system; VEGF has been shown to be expressed and control neural development in *Caenorhabditis elegans*, which lacks a vascular network (Popovici et al., 2002; Tarsitano et al., 2006). In the developing vertebrate brain, VEGF modulates axonal outgrowth (Sondell et al., 1999) and chemoattraction (Ruiz de Almodovar et al., 2011), and enhances dendritogenesis (Licht et al., 2010). Additionally, VEGF modulates synaptic activity of GABA in spinal neurons (Guerit et al., 2014) and glutamate in cerebellum granule cells (Meissirel et al., 2011). In the adult mammalian brain, VEGF can be induced to express in neurons (Hayashi et al., 1997; Lennmyr et al., 1998; Bao et al., 1999) and glial cells (Cobbs et al., 1998) in the brain following ischemic/hypoxic injury. Exogenous administration of VEGF in ischemic injured brains promotes neurogenesis (Jin et al., 2002) as well as increased differentiation, maturation, and neuritic outgrowth of newborn neurons (Wang et al., 2007, 2009) and astrocytic transformation into neurons (Shen et al., 2016). VEGF administration also provides neuroprotection in the brain after ischemic injury and in cultured neurons after hypoxic stimulation (Sun and Guo, 2005). Interestingly, VEGF can acutely modulate functions of potassium (Qiu et al., 2003; Xu et al., 2003) and calcium ion channels (Ma et al., 2009) on the neuronal membrane through activation of VEGF receptors in hippocampus in the brain slices and cultured neurons. VEGF-induced inhibition of ion channels causes decrease of neural activity and neuroprotection (Qiu et al., 2003; Wu et al., 2015). In summary, VEGF protein can express in neurons and have many direct roles in neural development and neuronal survival, as well as neural activity in axonal terminals. However, whether VEGF synthesized in soma can actively release and transport in the axon of neurons still need to be illustrative.

Recently, Foxton and his colleagues used intravitreal injection of VEGFR2 antibody and /or with AlexaFluor 488-conjugated cholera toxin B (CTB) in to Ins2^Akita^ diabetic and JR5558 spontaneous choroidal neovascularization (CNV) mice to look at the changes of anterograde transport in the retinal ganglion cells (RGCs). They interestingly reported that VEGFR2 antibody treated animal showed a distal transport loss in the superior colliculus prior to net RGC loss in the animals (Foxton et al., 2016). This phenomenon would be caused by two possibilities. (1) Reduction of endogenous VEGF receptor function may deteriorate neuronal damage and axonal degeneration. (2) Endogenous VEGF might have effect on the modulation of axonal trafficking. Therefore, direct cause of this phenomenon still needs to be investigated.

In general, proper neuronal function is determined by active axonal transport. Kinesin is one of the major microtubule-based motor proteins mainly responsible for anterograde axonal trafficking of various membrane-bounded organelles (MBO) along axons (Hirokawa and Takemura, 2005; Goldstein et al., 2008; Verhey and Hammond, 2009). Structurally, kinesin-1 is a tetrameric complex composed of two heavy chains a (kinesin-1A, -1B, and/or -1C, formerly KIF5A, -B, and/or -C) and two light chain (KLC1, KLC2) subunits (Brady, 1985; Vale et al., 1985; DeBoer et al., 2008; Hirokawa et al., 2010; Wang and Brown, 2010). Among them, kinesin-1B participates in axonal transport of BDNF (Butowt and von Bartheld, 2007; Colin et al., 2008) and prion protein vesicles (Encalada et al., 2011). Here we hypothesized that kinesin-1B and functional microtubules should participate in the axonal transport of VEGF if VEGF could be specifically trafficked in the axons.

Therefore, in the present study, we used primary hippocampal cultured neurons from Sprague-Dawley rat brains that were transfected with phVEGF-eGFP and kinesin-1B-DsRed vectors or kinesin-1B shRNA combined with confocal microscopic real-time imaging to detect fluorescent VEGF vesicles movement in axons of living cells. Our results provide direct evidence that VEGF synthesized in the neurons and participated in neural axonal transportation and transmission in hippocampal neurons.

## Materials and methods

### Primary hippocampal neuronal cultures and treatment protocols

#### Primary cultures

We prepared primary hippocampal neurons from 17 to 18-day-old embryonic brains of Sprague-Dawley (SD) rats according to the previously described procedures (Ma et al., 2009). All procedures involving animals were approved by the Medical Experimental Animal Administrative Committee of Shanghai and in accordance with the National Institute of Health Guide for the Care and Use of Laboratory Animals. The neurons were incubated at a density of 5 × 10^5^ cells/ml in a continuously humidified incubator with 5% CO_2_ and 95% O_2_ at 37°C with Neurobasal medium containing 2% B27 supplement.

#### Plasmids

Human VEGF_165_ cDNA was inserted into the pEGFP-N1 plasmid of green fluorescent protein between the constitutive cytomegalovirus promoter (pCMV) and eGFP (phVEGF-eGFP). Mouse kinesin-1B/KIF5B cDNA was inserted into the pDsRed2-N1 plasmid of red fluorescent protein between the pCMV and DsRed reporter gene (pm kinesin-1B -DsRed) using the GBclonart Seamless Assembly Kit (RockGene). Rat kinesin-1B shRNA or control shRNA were inserted into a lentivirus vector with a mCherry marker, which were provided by GeneChem (China). The shRNA sequence target kinesin-1B was ACAGCAGATCCAGAGTCACAGAGAA.

#### Transfection

The 7-day-old neuronal cultures were transfected with phVEGF-eGFP plasmid, or co-transfected with pm kinesin-1B -DsRed plasmids or shRNA plasmids using Lipofectamine 2000 reagent (Life Technologies, USA) to visualize axonal transport of VEGF. Only cells with DsRed fluorescence were imaged for VEGF-GFP and kinesin-B-DsRed vesicle transport. And only cells with mCherry fluorescence were imaged for VEGF-GFP vesicle transport.

Rat NRK-52E cells used for validation of kinesin-1B shRNA were cultured in DMEM containing 10% FBS at 37°C in 5% CO_2_. Cells were passaged and transfected with shRNA target constructs with Lipofectamine 2000.

#### Depolarization

To induce depolarization of the cellular membrane, we performed a KCl stimulation experiment based on a previously described protocol (Ma et al., 2009). Briefly, 10-day-old cultures in 35 mm dishes (1 × 10^6^ cells per dish) were rinsed three times with a balanced salt solution (BSS, pH 7.4) containing 130 mM NaCl, 5.4 mM KCl, 1.8 mM CaCl_2_, 5.5 mM glucose, and 20 mM HEPES. Then the cultures were incubated with 1 ml 60 mM KCl in stimulation solution (containing130 mM NaCl, 60 mM KCl, 1.8 mM CaCl_2_, 5.5 mM glucose, and 20 mM HEPES, pH 7.4) for 1 min, and the stimulation solution was collected for further analysis.

#### Nocodazole treatment

Cultured neurons at density of 1 × 10^6^ were treated with 0.1% DMSO (Vehicle) or 10 μM nocodazole (in neurobasal medium) for 30 min. Time-lapse images were acquired before and after treatment.

### Isolation of vesicles

SD rats (14-day-old) were deeply anesthetized and the hippocampus was rapidly removed for preparation of vesicles as previous described (Goldstein et al., 2008). The tissue was homogenized in isolated buffer (0.32 M sucrose, 0.5 mM EDTA, and 4 mM HEPES/NaOH pH7.4 containing a cocktail of protease inhibitors) and centrifuged at 480 × g and 20,000 × g for 5 and 15 min, respectively. The pellet was washed once and lysed in hypotonic medium (1 mM EDTA, 5 mM Tris/HCl, pH 7.4 and protease inhibitors) by homogenization, followed by incubation on ice for 45 min and centrifugation at 2,000 × g for 20 min. The supernatant was loaded underneath a discontinuous sucrose gradient of 1.2, 1.0, 0.8, 0.5, and 0.3 M sucrose in the buffer (0.5 mM EDTA, 4 mM HEPES/NaOH, pH 7.4), and centrifuged at 20,000 × g for 12–15 h. The interphase between 0.5 and 0.8 M was harvested. The samples were resuspended in isolation buffer and stored at −80°C for further analysis.

### Real-time RT-PCR

Total RNA was prepared from 10-day cultured neurons or 3-day transfected NRK-52E cells using Trizol reagent (Invitrogen) according to manufacture instructions. The mRNA (1 μg) of different samples was reversed transcribed to cDNA. Real-time PCR with SYBR Green detection was performed using Eppendorf Mastercycler ep realplex (Eppendorf, Germany). Glyceraldehyde-3-phosphate dehydrogenase (GAPDH) or β-actin was used as a control. The following primers were used: 5′-TGCACCCACGACAGAAGGGGA-3′ and 5′-TCACCGCCTTGGCTTGTCACAT-3′ for rat VEGF-A as previously described (Jin et al., 2000); 5′-GTCTTCCCCTCCATCGTGGG-3′ and 5′-TGGCTGGGGTGTTGA- AGGTC-3′ for β-actin; 5′-GCAAGACAGACGAGAACAAGC-3′ and 5′-GCCAAGTCCTGAACAAAGAGC-3′ for rat kinesin-1B; rat GAPDH primers were purchased from Haigene (China).

### Immunoblotting and immunoprecipitation

To detect VEGF, we performed immunoblotting assays with the samples obtained from hippocampal neurons. Proteins (10–20 μg) were separated on 10% sodium dodecyl sulfate-polyacrylamide gels and transferred onto PVDF membranes (Bio-Rad, Hercules, CA, USA). Rabbit anti-VEGF antibody (1:500, Santa Cruz Biotechnology) was used to detect VEGF. Horseradish peroxidase-conjugated goat anti-rabbit IgG (1:3000, Santa Cruz Biotechnology) served as the secondary antibody. Immunoreactivity was visualized using an enhanced chemiluminescence substrate system (ECL, Santa Cruz Biotechnology). Normalization was performed by stripping the blots and re-probing with a mouse monoclonal antibody specific for the β-isoform of actin (1:10,000, Sigma-Aldrich). Optical densities of immunostained bands were analyzed using an image processing and analysis system (Image J 2.0 software, NIH, Bethesda, MD, USA).

To detect biochemical interactions between the vesicle transporters of VEGF and kinesin-1B, we performed immunoprecipitation assays. A total of 200 μg protein of vesicles was incubated with rabbit monoclonal anti-kinesin-1B (anti-KIF5B) antibody (1:50, Abcam) or goat polyclonal anti-GFP antibody (1:50, Abcam) overnight at 4°C, followed by protein A (GE Healthcare Life Sciences) for 2 h at 4°C. The pellets were eluted with beta-mercaptoethanol and used for further immunoblotting assay with rabbit anti-KIF5B antibody (1:1,000, Abcam) or mouse monoclonal anti-VEGF antibody (1:500, Millipore), respectively.

To detect VEGF release in hippocampal neurons, we used immunoprecipitation to concentrate VEGF in the solution. The stimulation solution after KCl treatment was collected and incubated with rabbit polyclonal anti-VEGF antibody (5 μg anti-VEGF antibody was added to 1 ml stimulation solution, Santa Cruz) at 4°C overnight, followed by protein A beads (20 μl/reaction) at 4°C for 2 h. The pellets were eluted with beta-mercaptoethanol and used for further immunoblotting assay with mouse monoclonal anti-VEGF antibody (1:500, Millipore).

### Immunofluorescence and confocal microscopy

Seven-day cultured neurons were used for double immunofluorescent staining with mouse monoclonal anti-VEGF (1:200, Millipore) and rabbit polyclonal anti-MAP-2 antibodies (1:200, Millipore) or rabbit polyclonal anti-Synapsin I (Synapsin, 1:500, Millipore) at 4°C overnight, followed by incubation in 1:50 diluted anti-mouse IgG-FITC (Santa Cruz Biotechnology) or anti-rabbit IgG-Rhodamine (Santa Cruz Biotechnology) for 1 h at 37°C.

To detect VEGF vesicular localization, we smeared the isolated vesicles on glasses and performed immunofluorescent staining with mouse monoclonal anti-VEGF (1:200) and rabbit anti-Synapsin I (1:500) at 4°C for 4 h, and incubated in an antibody of donkey anti-mouse IgG-Alexa Fluor 488 (1:1000, Thermo Fisher Scientific) or donkey anti-rabbit IgG-Alexa Fluor 594 (1:1000, Thermo Fisher Scientific) for 1 h at 37°C to reveal fluorescent signals of VEGF and Synapsin I.

To detect exogenous expression of VEGF, we used immunostaining to detect eGFP expression in hippocampal neurons at 24 h after transfection of phVEGF-eGFP plasmids. The cultures were incubated with mouse monoclonal anti-eGFP (1:500) and rabbit polyclonal anti-MAP-2 (1:200) antibodies at 4°C overnight, followed by incubation in a 1:50 dilution of anti-mouse IgG-FITC (Santa Cruz Biotechnology) or anti-rabbit IgG-rhodamine (Santa Cruz Biotechnology) antibodies for 1 h at 37°C. To detect axonal expression of VEGF, the transfected cultures were incubated with a mouse monoclonal anti-SMI 312 antibody (1:500, Covance) at 4°C overnight, and incubated in second antibody of donkey anti-mouse IgG-Alexa Fluor 594 (1:1,000, Thermo Fisher Scientific) or donkey anti-mouse IgG-Alexa Fluor 647 (1:1,000, Thermo Fisher Scientific) for 1 h at 37°C. Fluorescent signals were detected at 535 nm excitation and emissions of 565 nm (rhodamine), 490 nm and 525 nm (FITC), 495 nm and 519 nm (Alexa Fluor 488), 590 nm and 617 nm (Alexa Fluor 594) and 652 nm and 668 nm (Alexa Fluor 647) by confocal laser-scanning microscopy (TCS SP5 or TCS SP8, Leica, Germany).

### Live cell confocal microscopy

We used live cell images to tract moving fluorescent signals in axons of cultured hippocampal neurons at 24 h after transfection with the phVEGF-eGFP fusion constructs under an inverted microscope (Leica SP5) with a 63 × oil objective equipped with a CCD camera at 488 nm excitation and 525 nm emissions. Images were taken from axons which were distinguished from dendrites according to morphology and showed thinner and longer than other neurites (Baas et al., 1988, 1989). Axons were traced from termini to the cell body and imaged within a region > 50 μm from the cell body. Time-lapse images were acquired by continuously capturing frames every 537 ms for 500 repeats at a resolution of 0.096 μm. Cultures were maintained in an incubator at 37°C during the imaging process.

To identify the relationship between microtubules and axonal transport of VEGF, dynamic images of the same axon were acquired before and 30 min after nocodazole treatment. Time-lapse images were acquired by continuously capturing frames every 537 ms for 150 repeats, at a resolution of 0.101 μm.

To identify biochemical interactions between kinesin-1B and VEGF vesicles in live hippocampal neurons, we co-transfected kinesin-1B-DsRed and phVEGF-eGFP vectors at a ratio of 1:3 into 7-day-old cultured hippocampal neurons. At 24 h after transfection, moving DsRed and GFP signals were real-time recorded in the axons at 535 nm excitation and 565 nm (DsRed) emissions, and 488 and 525 nm (eGFP). Time-lapse images were acquired by continuously capturing frames every 1.98 s for 100 repeats at a resolution of 0.0505 μm.

To verify participation of kinesin-1B in VEGF transport, we cotransfected phVEGF-eGFP with kinesin-1B shRNA or control shRNA constructs into 7-day-old cultured neurons. At 18–24 h after transfection, VEGF-eGFP movement in axons with mCherry fluorescence was acquired by continuously capturing frames every 537 ms for 200 repeats, at a resolution of 0.101 μm.

### Data analysis

#### Dynamic imaging analysis

The kymographs were generated by Image J 2.0 software to indicate moving signal trajectory (eGFP and DsRed signals) in axons during the period of real-time recording. All data were normalized according to time and length of the imaged axonal segment. Trajectories were classified as anterograde, retrograde, and stable (immobility), and two parameters describing transport dynamics were calculated: percent of cargo population and segmental velocity. A particle with a velocity >0.01 μm/s of the imaging period was defined as a moving particle (puncta), and its moving direction was read from the kymograph. A particle with a velocity <0.01 μm/s for the entire duration of the imaging period was defined as a stable (immobility) particle. Average velocity of each particle was calculated from the kymograph, and mean velocity and percent of cargo population of each experiment was calculated. Standard error was calculated per experiment. To determine the effect of nocodazole on axonal VEGF transport, data were quantified from at least three independent experiments and the statistical significance of differences between two groups (vehicle vs. nocodazole) was evaluated using the unpaired Student's *t*-test.

To determine VEGF level in hippocampal neurons after KCl treatment, data from five independent experiments were quantified and the statistical significance of differences between two groups was evaluated using the unpaired Student's *t*-test. All results are expressed as mean ± SEM. A difference was considered statistically significant at *P* < 0.05.

## Results

### VEGF is synthesized and stored in primary cultured hippocampal neurons

**Figure 1:**
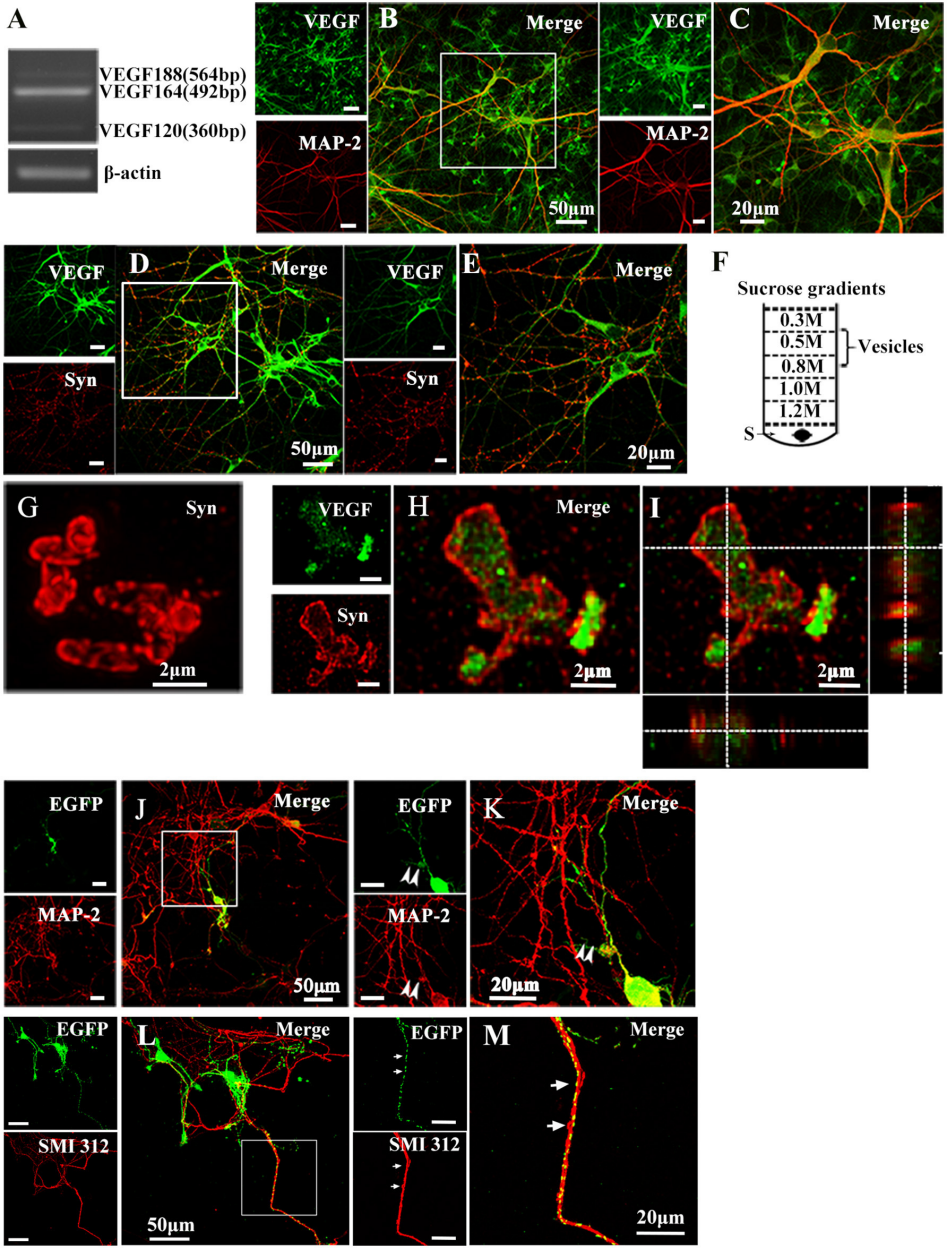
Representative images show VEGF expression and distribution in hippocampal neurons. **(A)** VEGF mRNA was detected by RT-PCR in primary hippocampal neuronal cultures (four independent experiments). **(B–E)** VEGF immunostaining in hippocampal neurons with neuronal marker MAP-2 **(B,C)** and Synapsin I (Syn, **D,E**). Panels **(C,E)** are magnified images of **(B,D)**, respectively. Scale bars = 50 μm **(B,D)**; 20 μm **(C,E)**. **(F)** Schematic diagram of a vesicle extraction experiment showing the 0.5–0.8 M fraction used as starting material for vesicle identification in **(G)** and **(H)**. Supernatant of lysed synaptosomes (S) obtained from rat hippocampi homogenate was bottom-loaded. Buffers did not contain detergents to prevent membrane disruption. **(G)** Identification of isolated vesicles by immunostaining with Synapsin I. Scale bars = 2 μm. **(H,I)** Double-immunostaining with Synapsin I and VEGF in isolated vesicles from rat hippocampi. **(I)** Orthogonal view of a confocal z-stack illustrates VEGF expression in Synapsin I-positive vesicles. **(J,K)** VEGF-eGFP expression in hippocampal neurons at 24 h after transfection of the VEGF-eGFP plasmid. Immunostaining of eGFP (green) and MAP-2 (red) show VEGF expression in MAP-2-negative axonal branches (**K**, magnified images of **J**). Scale bars = 50 μm **(J)**; 20 μm **(K)**. **(L,M)** VEGF-eGFP expression in axon at 24 h after transfection of VEGF-eGFP plasmid. Immunostaining of SMI 312 (red) show VEGF expression in SMI 312-positive axon (**M**, magnified images of **L**). Scale bars = 50 μm **(J)**; 20 μm **(K)**.

To determine whether cultured hippocampal neurons synthesize VEGF, we measured VEGF mRNA expression in neurons from 10-day-old cultures using reverse transcription PCR (RT-PCR) analysis. Consistent with previous studies (Jin et al., 2000), results revealed neuronal mRNA expression of VEGF120, VEGF164, and VEGF188 mRNA, three alternative splicing isoforms. Among them, VEGF164 mRNA levels were greatest (Figure 1A). To detect cellular distribution of VEGF in hippocampal neurons, double-immunostaining was performed with antibodies specific to VEGF and MAP-2, a mature neuronal marker, or Synapsin I, a vesicle marker. As shown in Figures 1B–E, VEGF was co-expressed with MAP-2 (VEGF^+^-MAP-2^+^, Figures 1B,C) and Synapsin I (VEGF^+^-Synapsin I^+^, Figures 1D,E) in the hippocampal neurons.

To determine whether VEGF is stored in vesicles, vesicles were isolated from rat hippocampi through a discontinuous gradient sucrose. Vesicles harvested from the 0.5-M to 0.8-M sucrose gradients (Figure 1F) were verified by immunostaining with Synapsin I (Figure 1G). Using the same vesicles samples, double-staining of VEGF and Synapsin I was performed to detect VEGF expression in vesicles of hippocampal neurons (Figures 1H,I).

### VEGF is transported in axons of hippocampal neurons

**Figure 2:**
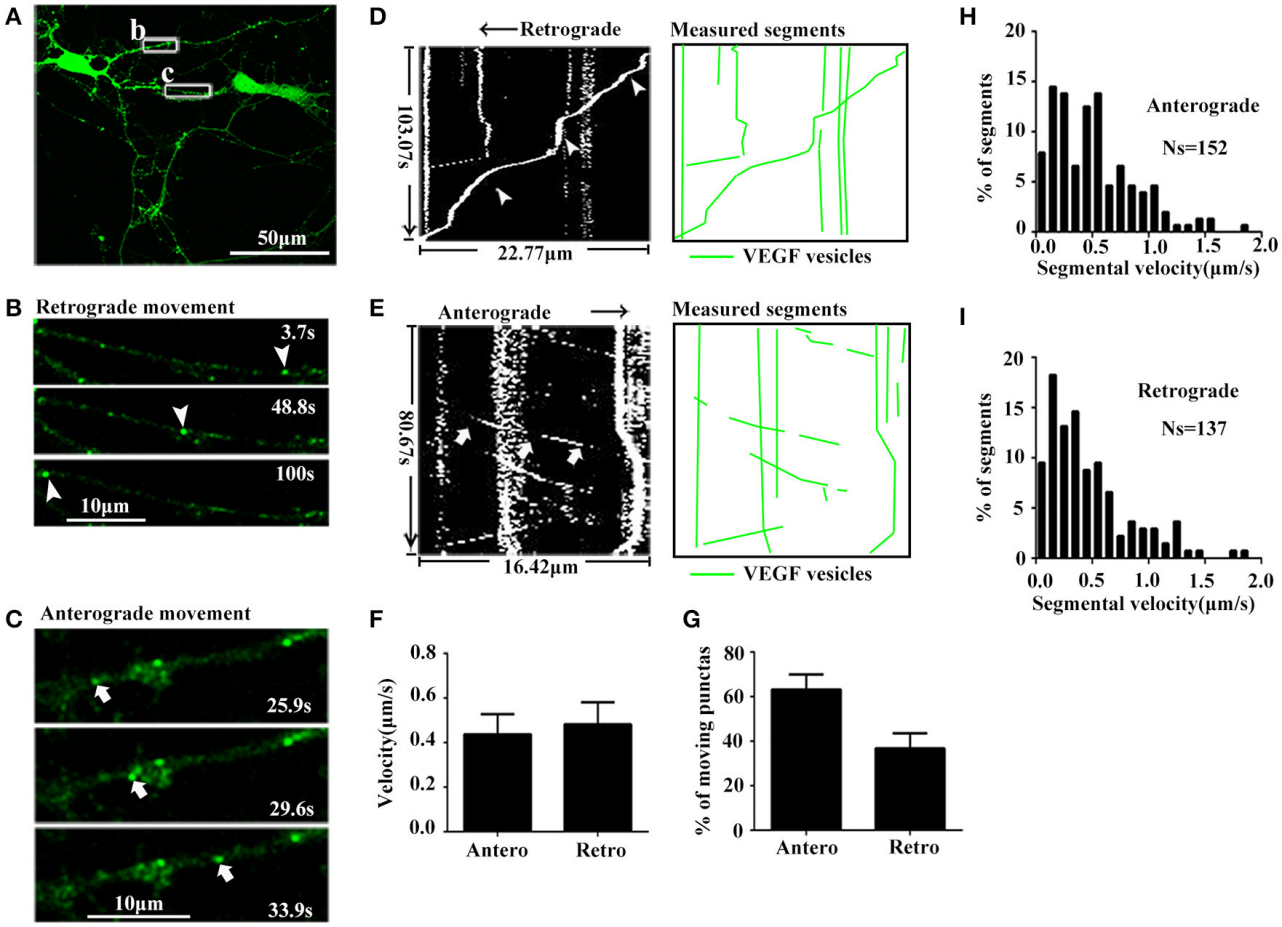
Dynamic images show retrograde and anterograde transport of VEGF. **(A)** Representative images of axon region (box b and c). Scale bar = 50 μm. **(B)** Magnified images of box b in **(A)**, and dynamic images show retrograde-moved puncta (indicated by arrowheads). Scale bar = 10 μm. **(C)** Magnified images of box c in **(A)**, and dynamic images show anterograde-moved puncta (indicated by arrows). Scale bar = 10 μm. **(D)** Kymograph of VEGF movement in box b; line indicated by arrowheads is the moving trajectory of a punta indicated by arrowheads in **(B)**. Measured segments of VEGF were masked by green lines. **(E)** Kymograph of VEGF movement in box c; line indicated by arrows is the moving trajectory of a punta indicated by arrows in **(C)**. **(F)** Accumulative data of axonal transport velocity of VEGF from four independent experiments. **(G)** Quantification of percentages of anterograde (Antero) punctas and retrograde (Retro) punctas among total moving punctas in four independent experiments. **(H,I)** Histograms for segmental velocity of anterograde transported punctas **(H)** and retrograde transport punctas **(I)**. Ns means the number of segments. Data represent mean ± S.E.M.

As mentioned above, results showed that VEGF was synthesized in neurons and stored in vesicles. Therefore, we next analyzed whether neuronal VEGF was transported in the neuronal axons. To visualize axonal movement of VEGF in live neurons, we transfected 7-day-old hippocampal neuronal cultures with phVEGF-eGFP vectors to detect eGFP signals in neurons, as indicated by double-staining of MAP-2 and eGFP (Figure 1J). Our results showed that VEGF expression was detected in the soma, dendrites, and MAP-2^−^ axonal branches as indicated by arrowheads in Figure 1K. To further elucidate axonal expression of VEGF, transfected cultures were also immunostained with axonal specific marker, SMI 312 (Ulfig et al., 1998). As shown in Figures 1L,M, VEGF-eGFP signals were detected in SMI 312 positive axon, that distinguished from dendrites which are thinner in diameter and longer. We then performed time-lapse imaging under a confocal laser-scanning microscope and traced eGFP fluorescent signal movement in the axons of live hippocampal neurons. Based on subsequently described methods, we chose a healthy transfected neuron (Figure 2A) and recorded eGFP signals for imaging analysis at 37°C [Figures 2B,C, also shown in the animations (Supplementary Movies 1, 2)]. Results from the dynamic images showed that some eGFP signals moved quickly in axons of living cells in anterograde and retrograde directions. Figures 2B,C show retrogradely and anterogradely moving puncta, as indicated by arrowheads and arrows in the axon branches, respectively. To characterize the properties of axonal VEGF transport in neurons, we further analyzed imaging data with Image J 2.0 software to generate 2D kymographs. The kymographs in Figures 2D,E were generated from imaging data corresponding to Figures 2B,C, showing that most punctate moved retrogradely or anterogradely, and some were immobile.

Quantification data showed that among the total moving punctate, 63.2 ± 6.81% moved anterogradely, and 36.8 ± 6.81% moved retrogradely (*n* = 4, Figure 2G). Both anterograde and retrograde transport punctates exhibited a wide velocity range. Maximal velocities of anterograde and retrograde transport punctate were 1.83 μm/s (*n* = 152 segments, Figure 2H) and 1.86 μm/s (*n* = 137 segments, Figure 2I), respectively. Mean velocities of anterograde and retrograde VEGF transport were 0.44 ± 0.090 and 0.48 ± 0.099 μm/s (*n* = 4, Figure 2F). These results demonstrated that VEGF could be bidirectionally transported in the axons of hippocampal neurons.

### Nocodazole inhibits VEGF trafficking in axons of hippocampal neurons

To determine whether microtubules participate in axonal trafficking of VEGF, we observed changes in axonal VEGF transport before and 30 min after treatment with vehicle or nocodazole, an inhibitor of microtubules.

**Figure 3:**
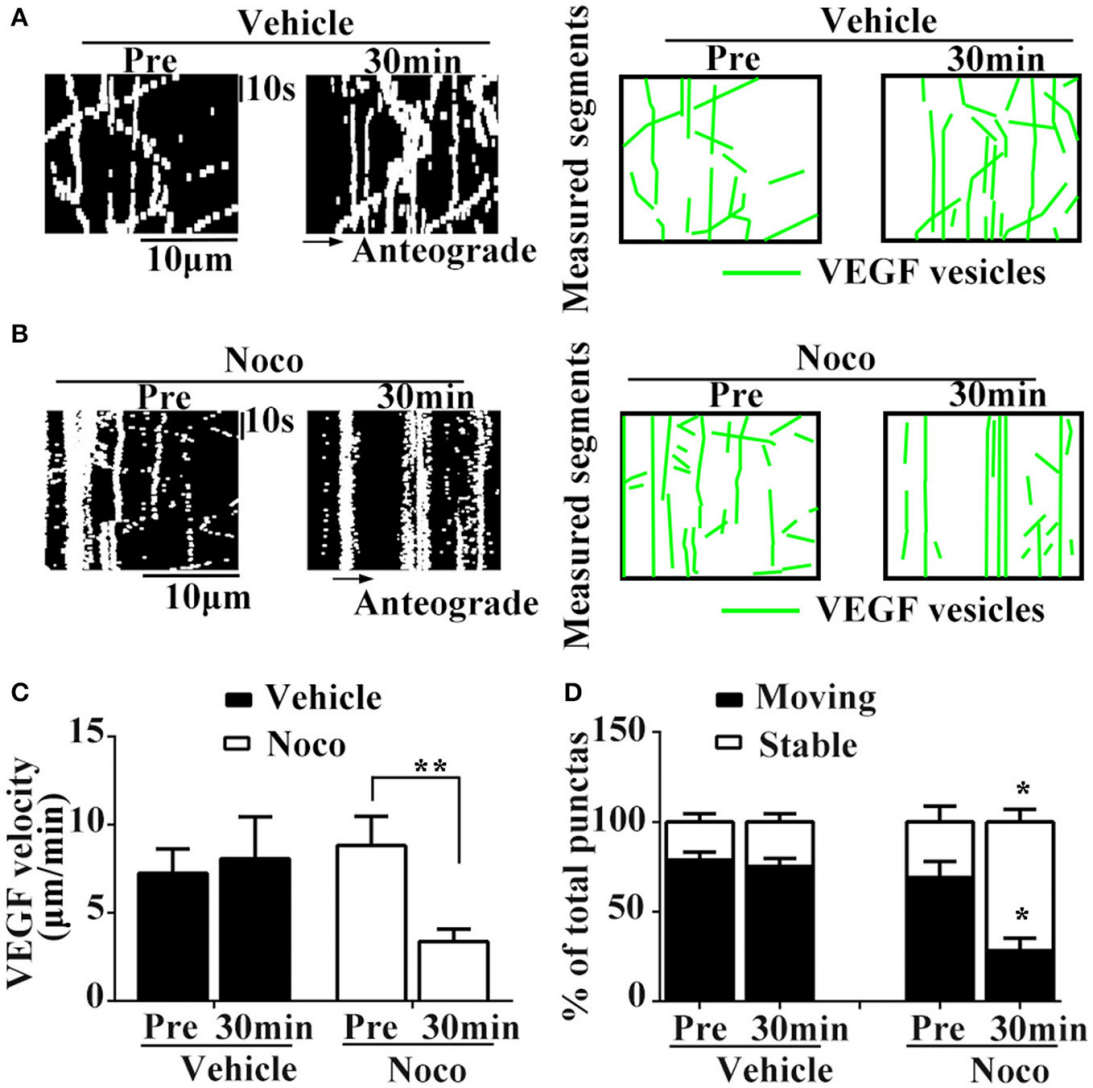
Dynamic images show microtubules-dependent axonal transport of VEGF. **(A,B)** Representative kymographs of VEGF transport in vehicle group **(A)** and nocodazole (Noco, 10 μM, final concentration) treatment group **(B)**. The size of kymographs is 20 μm (distance; scale bars = 10 μm) and 120 s (time; scale bars = 10 s). Measured segments of VEGF were masked by green lines. **(C)**, Accumulative data of transport velocity of VEGF before and 30 min after vehicle or nocodazole treatment from six or four independent experiments. **(D)**, Accumulative data of the percentage of moving punctas and immobility punctas before and 30 min after vehicle or nocodazole treatment from three independent experiments. Data represent mean ± S.E.M, ^**^P < 0.01, ^*^P < 0.05.

Results showed that nocodazole post-treatment significantly reduced moving eGFP signals and increased stable/immobile eGFP signals in the axons (Figures 3A–D). Post-treatment of nocodazole significantly reduced the percentage of moving eGFP signals from 69.1 ± 15.36% to 28.3 ± 12.08% of pretreatment (*P* = 0.011, *n* = 3, Figure 3D), and increased the percentage of immobile particles from 30.9 ± 15.36% to 71.7 ± 12.08% (*P* = 0.01, *n* = 3, Figure 3D). Additionally, post-treatment with nocodazole significantly reduced axonal transport velocity of VEGF (3.8 ± 0.69 μm/min) compared with pretreatment (8.8 ± 1.67 μm/min), as shown in Figure 3C (*P* = 0.012, *n* = 4). Moreover, vehicle treatment did not alter the percentage of immobile particles (24.9 ± 4.57% vs. 21.3 ± 4.47% in pretreatments, *P* = 0.305, *n* = 3) or transport velocity (8.1 ± 2.36 μm/min vs. 7.3 ± 1.37 μm/min in pretreatments, *P* = 0.384, *n* = 6, Figures 3C,D). These results suggested that axonal VEGF transport depends on the presence of functional microtubules in neurons.

### Kinesin-1B co-transports with VEGF vesicles in axons of hippocampal neurons

As previously mentioned, VEGF transport in axons of hippocampal neurons required functional microtubules, which serves as an axonal transporting cargo. It is well-known that kinesin-1B is one of the microtubule-based complex motors that drives some vesicles movements in axons. Therefore, in the next step, we analyzed whether kinesin-1B is involved in the VEGF transport cargoes.

**Figure 4:**
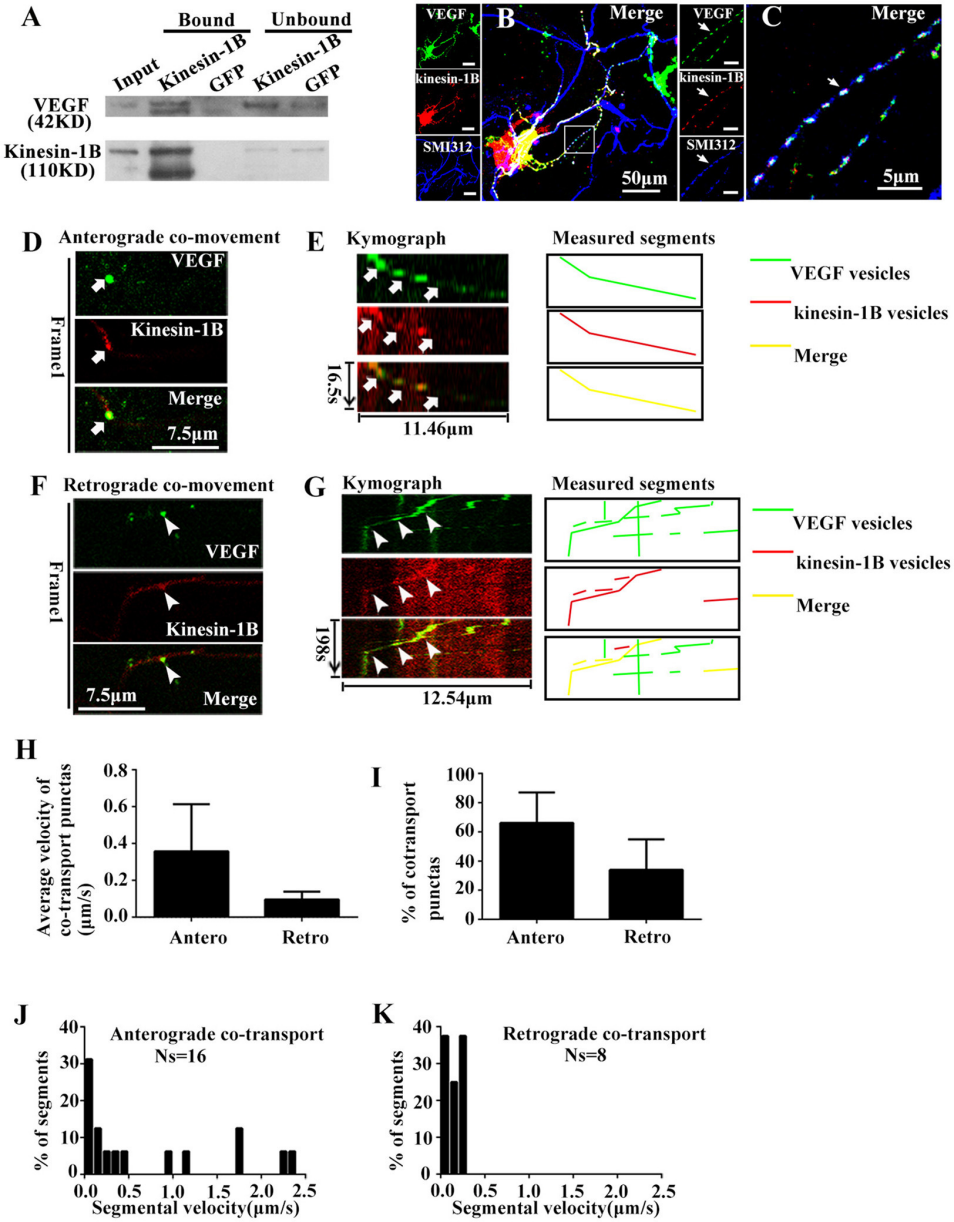
Kinesin-1B associates with vesicular transport of VEGF. **(A)** Biochemical association of kinesin-1B with VEGF vesicles. An antibody specific for kinesin-1B was used to pull-down associated membrane components from isolated vesicles, including VEGF vesicles (indicated in Figure 1F). Anti-GFP antibody was used as a control. The bead-bound (bound) and unbound fractions (unbound) were analyzed by immunoblotting with antibodies specific for the indicated proteins. **(B,C)** Simultaneous transfection of VEGF-eGFP plasmids and kinesin-1B -DsRed plasmids at 5 DIV (days *in vitro*), and distributions of VEGF-eGFP and kinesin-1B -DsRed were detected by immunostaining with axonal specific marker SMI 312 at 48 h after transfection. The representative images show VEGF and kinesin-1B co-localization in SMI 312 positive axon, as indicated by arrows in **(C)**. Panel **(C)** is magnified images of box in **(B)**. Scale bars = 50 μm **(B)**; 5 μm **(C)**. **(D–G)** Anterograde **(D,E)** and retrograde **(F,G)** co-transport of VEGF and kinesin-1B. The first frame **(D,F)** and kymograph **(E,G)** of VEGF (green in merged image) and kinesin-1B (magenta in merged image) are provided. Time (in seconds) and distance (in micrometers) are labeled on merged kymographs. Measured segments were masked by lines with different colors, green lines represented as VEGF vesicles, red lines represented as kinesin-B vesicles, yellow lines represented as merged vesicles. Scale bars = 7.5 μm **(D,F)**. **(H)** Accumulative data of average velocity of co-transported puntas from four or three independent experiments. **(I)** Quantification of the percentages of anterograde and retrograde co-transported punctas among total co-transported punctas in four independent experiments. **(J,K)** Histograms for segmental velocity of anterograde co-transported punctas **(J)** and retrograde co-transported punctas **(K)**. Ns means the number of segments. Antero and Retro represent anterograde and retrograde, respectively. Data represent mean ± S.E.M.

First, immunoprecipitation was performed with vesicles fractions to determine whether kinesin-1B is biochemically associated with VEGF vesicles. An antibody against kinesin-1B was used to pull-down the associated components, which was further immunoblotted with VEGF and kinesin-1B antibodies. Results showed that VEGF was immunoprecipitated by kinesin-1B (Figure 4A). An antibody specific to GFP was used as a negative control in this co-immunoprecipitation experiment. Because VEGF is in the vesicular lumen, reverse immunoprecipitation using VEGF antibodies to pull-down kinesin-1B was not possible without breaking vesicular membranes. Therefore, results from present data suggest that kinesin-1B is biochemically associated with VEGF vesicles.

Co-localization of eGFP and DsRed fluorescence was imaged in fixed hippocampal neurons at 48 h after co-transfection with phVEGF-eGFP and kinesin-1B-DsRed vectors to determine whether kinesin-1B interacts with VEGF vesicles. Either eGFP or DsRed fluorescence revealed punctates, which represent typical vesicle morphology, and most were colocalized in the SMI 312 positive axons (Figures 4B,C), suggesting morphological co-distribution in the axons. Therefore, we further performed two-color time-lapse imaging with co-transfected neurons to determine co-trafficking of VEGF and kinesin-1B in the axons. Results showed that about half of VEGF punctate signals (59.3% in 54 eGFP punctas) were singly isolated in the axons, and most kinesin-1B punctate signals were colocalized with VEGF in the axons (91.7% in 24 DsRed punctas), although to a lesser amount compared with VEGF signals. Double-color punctates in the axons were selected for further investigation. Results showed bidirectional co-trafficking of VEGF and kinesin-1B in axons [Figures 4D–G, also shown in the animations (Supplementary Movies 3, 4)].

Quantification results showed that mean velocities of anterograde and retrograde co-transporting in axons were 0.36 ± 0.256 μm/s (*n* = 4) and 0.10 ± 0.043 μm/s (*n* = 3), respectively (Figure 4H). Among the total co-transported punctas, the percentages of anterograde and retrograde moving punctate were 66.1 ± 20.90% and 33.9 ± 20.91% (*n* = 4, Figure 4I), respectively. The moving velocity of anterograde transporting punctas exhibited a wider range than the retrograde co-trafficked punctas. Maximal velocity of anterograde co-transport was 2.31 μm/s (*n* = 16 segments, Figure 4J), while maximal velocity of retrograde co-transport was 0.28 μm/s (*n* = 8 segments, Figure 4K). These results indicate that kinesin-1B was co-transported along with VEGF vesicles in the axons of hippocampal neurons.

### Kinesin-1B shRNA reduces axonal transpot of VEGF in the hippocampal neurons

Having shown that kinesin-1B interact with VEGF vesicles, we next tested whether these physical interactions translated into functional transport requirements. Thus, we tested functional changes of VEGF transport in hippocampal neurons co-transfected with VEGF-eGFP and kinesin-1B shRNA-mCherry constructs, which reduced protein expression of kinesin-1B by 67.3 ± 8.47% (vs. control shRNA, *n* = 3).

**Figure 5:**
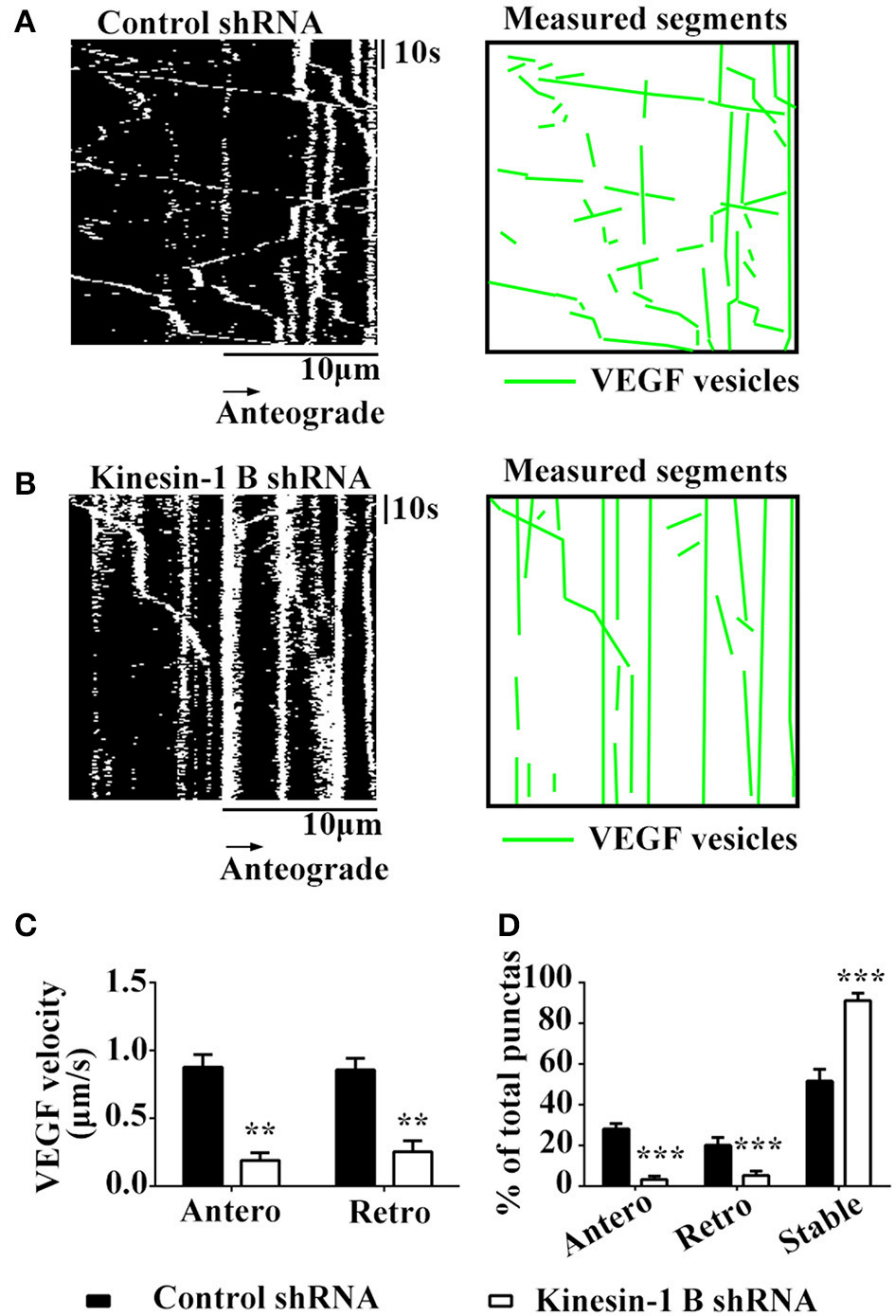
Kinesin-1B is required for bidirectional transport of VEGF in hippocampal axons. **(A,B)** Representative kymographs of VEGF transport in control shRNA group **(A)** and kinesin-1B shRNA group **(B)**. The size of kymographs is 20 μm (distance; scale bars = 10 μm) and 104 s (time; scale bars = 10 s). Measured segments of VEGF were masked by green lines. **(C)** Accumulative data of transport velocity of VEGF vesicles in anterograde and retrograde directions from five independent experiments. **(D)** Accumulative data of the percentage of anterograde, retrograde and stable/immobile vesicles from five independent experiments. Antero and Retro represent anterograde and retrograde, respectively. Data represent mean ± S.E.M, ^***^P < 0.001, ^**^P < 0.01.

Results showed that kinesin-1B shRNA treatment caused significant reduction of moving VEGF-eGFP signals and increases of stable/immobile VEGF-eGFP signals in the axons (Figures 5A–D). Kinesin-1B shRNA treatment significantly decreased the velocities both in anterograde and retrograde transport of VEGF vesicles (Figures 5A–C). Kinesin-1B shRNA treatment reduced the velocity of anterograde transport of VEGF vesicles to 0.19 ± 0.056 μm/s and retrograde to 0.26 ± 0.079 μm/s compared with control shRNA treatment (0.88 ± 0.092 μm/s, *P* = 0.0026, and 0.86 ± 0.085 μm/s, *P* = 0.0067, respectively, *n* = 5, Figure 5C). Moreover, kinesin-1B shRNA treatment significantly decreased the percentages of anterograde and retrograde transport of VEGF vesicles compared with control shRNA treatment (anterograde transport, 3.4 ± 1.55% vs. 28.2 ± 2.71%, *P* = 0.00029; retrograde transport, 5.4 ± 2.16% vs. 20.2 ± 3.77%, *P* = 0.0069; *n* = 5 per group, Figure 5D), and increased the percentage of immobile VEGF vesicles (91.2 ± 3.66% vs. 51.7 ± 5.91% in control shRNA treatment, *P* = 0.00089, *n* = 5, Figure 5D). These results suggest that axonal VEGF transport requires the presence of kinesin-1B in neurons.

### VEGF release from hippocampus neurons

**Figure 6:**
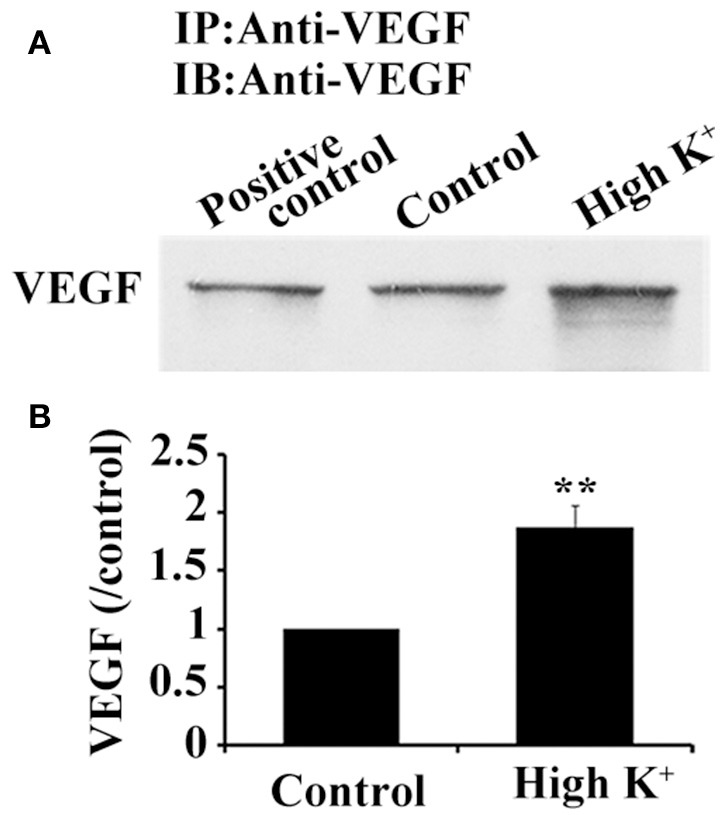
High K^+^ stimulation increases VEGF release from neurons. **(A)** Immunoprecipitation of VEGF protein in supernatant from primary cultures of hippocampal neurons. Cell lysates were used as positive controls. **(B)** Quantitative analysis of VEGF expression in supernatant from primary cultures of hippocampal neurons in five independent experiments. Data represent mean ± S.E.M, ^**^P < 0.01.

Since synthesized VEGF in neurons was stored in vesicles and bidirectionally trafficked in axons *via* functional microtubule-based moving cargoes, we then analyzed whether VEGF vesicles, which were anterogradely transported from the soma to the terminals, were actively released. Cultured neurons were acutely treated with potassium chloride (high K^+^) at a final concentration of 60 mM for 1 min to depolarize the neuronal membrane; VEGF was then detected in the treated medium. VEGF levels were concentrated by using VEGF-specific antibody to precipitate VEGF in the treated medium, followed by immunoblotting. Results showed significantly increased VEGF levels in the high K^+^-treated group compared with the vehicle group (control, *P* = 0.003, *n* = 5), as shown in Figure 6.

## Discussion

Results from the present study provide the first and direct evidence that neuronal VEGF is specifically transported in axons and actively released from neurons. We used live cell imaging and time-lapse microscopy to track axonal VEGF transport in hippocampal neurons transfected with phVEGF-eGFP vectors, revealing that neuronal VEGF stored in vesicles and could be anterogradely and retrogradely transported in axons. Co-transfected with kinesin-1B-DsRed vectors, we found that vesicle VEGF morphologically and biochemically interacted with kinesin-1B and co-trafficking in the axons. With treatment of kinesin-1B shRNA or microtubules inhibitor nocodazole, we found effective axonal trafficking of VEGF vesicles depended on the presence of functional microtubule and motor protein kinesin-1B. Moreover, the vesicle VEGF could be released from the neurons by acute depolarizing stimulation. The present results extend our knowledge that VEGF participates in neural axonal transportation and transmission besides its vascular biological effects. These results help us to understand why VEGF is important for the maintenance of neuronal activity and plasticity in mammalian brains throughout life.

Several lines of evidence have demonstrated that VEGF plays a neurotrophic effect during development (Sondell et al., 1999; Licht et al., 2010; Erskine et al., 2011; Ruiz de Almodovar et al., 2011) and in the adult brain (Sun et al., 2003; Sun and Guo, 2005; Wang et al., 2009). In general, the neurotrophic effects on neurons rely on effective axonal trafficking. In the present study, we hypothesized that VEGF was synthesized in neurons and transported in axons. We used a variety of approaches to test this hypothesis. PCR results show that VEGF is expressed in neurons, and immunostaining (Figure 1) and immuno-isolation of vesicles (Figure 3) showed that VEGF is stored in vesicles. VEGF-eGFP vesicle signals are anterogradely and retrogradely transported through axons (Figure 2), as indicated by real-time recorded trafficking signals in living neurons following transfection with VEGF-eGFP vectors (Figure 2). As previously known, active axonal transport should exhibit specific characteristics, which include the following: (1) specific property of vesicle transport in the axon; (2) microtubule-based axonal trafficking; and (3) participation of motor proteins in vesicle transport. Interestingly, axonal transport of VEGF vesicles was specific, because the characteristics of trafficking were determined by the following results. First, the trafficking speed of VEGF vesicles in axons (Figure 2) is comparable to the reported value of axonal transport of synaptophysin (mean ± SD, 0.69 ± 0.33 μm/s) (Nakata et al., 1998) and BDNF (mean ± SEM, 0.3 ± 0.1 μm/s) (Kohara et al., 2001). Second, axonal transport of VEGF vesicles requires the presence of functional microtubules in the neurons, since the capacity of transport was reduced by treatment with nocodazole (Figure 3). Third, axonal transport of VEGF vesicles relies on the depend presence of motor protein kinesin-1B as shown in the following evidences. (1) kinesin-1B biochemically bound to VEGF vesicles, because kinesin-1B was able to pull-down the VEGF-positive vesicle membrane (Figure 4A) and co-stained with VEGF vesicles in axons of fixed neurons (Figures 4B,C). (2) kinesin-1B and VEGF signals were co-trafficking in the axons evidenced by two-color time-lapse imaging analysis (Figures 4D–G). (3) Treatment of kinesin-1B shRNA knockdown protein expression of kinesin-1B and caused reduction of moving speed of axonal transport of VEGF (Figure 5). Taken together, the results clearly and directly suggest that VEGF specifically transported in neuronal axons depending on the presence of functional microtubules and axonal trafficking motor kinesin-1B. As we have known, functional axonal transport relates to effective neurotransmission and neuron survival. Therefore, the present results of VEGF axonal transport in the neuron could help to explain the mechanism of VEGF's neuroprotection and neural plasticity in the brains. These findings also provided first and direct evidence to support the propose as previous discussed (Foxton et al., 2016).

Interestingly, most of co-trafficking puncta of VEGF vesicles, which were associated with kinesin-1B, were shown to move in an anterograde pattern, and some of them moved very rapidly (Figure 4J). However, very few exhibited slow retrograde transport. Inhibition of kinesin-1B by shRNA leads to diminished motility in both directions (Figure 5). These data suggest that kinesin-1B may preferentially favor anterograde transport of VEGF vesicles but also plays a vital role in retrograde transport of VEGF vesicles. Also retrograde and anterograde motility indicated that different kinesins and dynein may be involved in VEGF trafficking and kinesin-1B may be involved in bidirectional transport. This phenomenon is beyond of traditional “tug-of-war” paradigm, which is called “paradox of codependence” by Hancock (2014). This phenomenon is also reported by numerous knockout and inhibition studies in a variety of systems (Goldberg, 1982; Brady and Pfister, 1991; Waterman-Storer et al., 1997; Martin et al., 1999; Haghnia et al., 2007; Barkus et al., 2008; Ally et al., 2009; Encalada et al., 2011; Moughamian and Holzbaur, 2012), and further studies are needed to clarify its mechanism. In addition, our study is consistent with previous a report showing that kinesin-1 is responsible for anterograde axonal transport of BDNF, an important neurotrophin (Colin et al., 2008). Broad range of velocities of VEGF trafficking is also indicative for different motors attached to VEGF vesicle. Both kinesin heavy chain variants kinesin-1A and kinesin-1B moved in fast anterograde transport, but kinesin-1A moved at 5–6 times the rate of kinesin-1B. As previously reported, kinesin-1A is enriched on small tubulevesicular structures like synaptic vesicles and kinesin-1B is predominantly on mitochondria (Elluru et al., 1995). Therefore, future studies to clarify the role of kinesin-1A in VEGF trafficking may be helpful to better understanding of molecular mechanisms of VEGF trafficking.

Previous studies have proposed that VEGF acts as neurotrophin based on the neuroprotective and neural repair effects in ischemic or traumatic neuronal injury models *in vivo* and *in vitro* (Sun et al., 2003; Ma et al., 2009, 2011), as well as its neurogenic effects in developing and adult brains under physiological and pathological conditions (Jin et al., 2002; Louissaint et al., 2002; Wang et al., 2007). VEGF also exhibits biological characteristics for neuromodulator/neuropeptide in neurons. Results from the present study indicate that VEGF is synthesized in neurons (Figure 1A) and stored in vesicles (Figures 1H,I). VEGF also co-transports with microtubule-based motor kinesin-1B from the soma to the distal axon of neurons (Figures 4, 5). More importantly, neuronal VEGF could be induced to release rapidly by acute depolarization stimulation (Figure 6). Additionally, VEGF receptors (Flt and Flk) are expressed on neuronal membranes, which can be rapidly and reversibly activated, thereby modulating neurotransmission (McCloskey et al., 2005; Kim et al., 2008) and electrophysiological activity of the neuronal membrane (McCloskey et al., 2008) via regulation of multiple ion channels (Xu et al., 2003; Ma et al., 2009). Putting all together, VEGF in the neuron showed some properties of neuromodulator/neuropeptide. In the brain, neuronal viability and proper functioning is related to active neurotransmission. Therefore, future studies to determine the mechanisms of VEGF-mediated neurotransmission may help for the development of novel therapeutic strategies to improve neuroprotection against noxious injury or neurodegeneration.

## Author contributions

PY: Conception and design; collection and/or assembly of data; data analysis; manuscript writing. XS: Collection and/or assembly of data. Z-WK: Collection and/or assembly of data. K-WW: Data analysis. Y-LH: Collection of data. F-YS: Conception and design, data analysis and interpretation; manuscript writing; final approval of manuscript.

### Conflict of interest statement

The authors declare that the research was conducted in the absence of any commercial or financial relationships that could be construed as a potential conflict of interest.

## Supplementary material

Supplementary Movie 1 Axonal transport of VEGF-eGFP vesicles in hippocampal axon, related to Figure 2B. A video of VEGF-eGFP vesicle transport in a 9-day-old hippocampal axon. Cell body is toward the left, terminus is toward the right. Movie was taken at 0.67 s/frame, for 15 s.

Supplementary Movie 2 Axonal transport of VEGF-eGFP vesicles in hippocampal axon, related to Figure 2C. A video of VEGF-eGFP vesicle transport in a 9-day-old hippocampal axon. Cell body is toward the left, terminus is toward the right. Movie was taken at 0.67 s/frame, for 154 s.

Supplementary Movie 3 Anterograde co-transport of VEGF-eGFP vesicles and kinesin-1B-DsRed particles in hippocampal axon, related to Figure 4D. A video of VEGF-eGFP vesicle transport in a 7-day-old hippocampal axon. VEGF-eGFP is green, and kinesin-1B-DsRed is red. Cell body is toward the left, terminus is toward the right. Movie was taken at 1.83 s/frame, for 15 s.

Supplementary Movie 4 Retrograde co-transport of VEGF-eGFP vesicles and kinesin-1B-DsRed particles in hippocampal axon, related to Figure 4F. A video of VEGF-eGFP vesicle transport in a 7-day-old hippocampal axon. VEGF-eGFP is green, and kinesin-1B-DsRed is red. Cell body is toward the left, terminus is toward the right. Movie was taken at 1.98 s/frame, for 196 s.

## Abbreviations

BSS: balanced salt solution
CMV: constitutive cytomegalovirus promoter
GABA: ⋎-aminobutyrate
GAPDH: glyceradehyde-3-phosphate dehydrogenase
GFP: green fluorescent protein
MAP-2: microtubule associated protein 2
MBO: membrane-bounded organelles
VEGF: vascular endothelial growth factor
SD: Sprague-Dawley
Syn: synapsin-I.
